# Prediction of survival in infants with congenital diaphragmatic hernia and the response to inhaled nitric oxide

**DOI:** 10.1007/s00431-022-04568-8

**Published:** 2022-07-28

**Authors:** Fahad M. S. Arattu Thodika, Svilena Dimitrova, Mahesh Nanjundappa, Mark Davenport, Kypros Nicolaides, Theodore Dassios, Anne Greenough

**Affiliations:** 1 Department of Women and Children’s Health, School of Life Course Sciences, Faculty of Life Sciences and Medicine, King’s College London, London, UK; 2 Neonatal Intensive Care Centre, King’s College Hospital NHS Foundation Trust, London, UK; 3 Department of Paediatric Surgery, King’s College Hospital NHS Foundation Trust, London, UK; 4 Fetal Medicine Unit, King’s College Hospital NHS Foundation Trust, London, UK; 5 NIHR Biomedical Research Centre Based at Guy’s and St Thomas NHS Foundation Trust and King’s College London, London, UK

**Keywords:** Congenital diaphragmatic hernia, Inhaled nitric oxide, Oxygenation index, PaO_2_/FiO_2_ ratio

## Abstract

The use of inhaled nitric oxide (iNO) in treating pulmonary hypertension in infants with congenital diaphragmatic hernia (CDH) is controversial. Our aims were to identify factors associated with survival in CDH infants and whether this was influenced by the response to iNO. Results of CDH infants treated in a tertiary surgical and medical perinatal centre in a ten year period (2011–2021) were reviewed. Factors affecting survival were determined. To assess the response to iNO, blood gases prior to and 30 to 60 min after initiation of iNO were analysed and PaO_2_/FiO_2_ ratios and oxygenation indices (OI) calculated. One hundred and five infants were admitted with CDH; 46 (43.8%) infants died. The CDH infants who died had a lower median observed to expected lung to head ratio (O/E LHR) (*p* < 0.001) and a higher median highest OI on day 1 (HOId1) (*p* < 0.001). HOId1 predicted survival after adjusting for gestational age, Apgar score at 5 min and O/E LHR (odds ratio 0.948 (95% confidence intervals 0.913–0.983)). Seventy-two infants (68.6%) received iNO; 28 survived to discharge. The median PaO_2_ (46.7 versus 58.8 mmHg, *p* < 0.001) and the median PaO_2_/FiO_2_ ratio (49.4 versus 58.8, *p* = 0.003) improved post iNO initiation. The percentage change in the PaO_2_/FiO_2_ ratio post iNO initiation was higher in infants who survived (69.4%) compared to infants who died (10.2%), *p* = 0.018.

*Conclusion*: The highest OI on day 1 predicted survival. iNO improved oxygenation in certain CDH infants and a positive response was more likely in those who survived.

**Table:** 

**What is Known:** *• The use of iNO is controversial in infants with CDH with respect to whether it improves survival.*	
**What is New:** *• We have examined predictors of survival in CDH infants including the response to iNO and demonstrated that the highest oxygenation index on day 1 predicted survival (AUCROC =0.908).* *• Certain infants with CDH responded to iNO and those with a greater response were more likely to survive.*

**What is Known:**

*• The use of iNO is controversial in infants with CDH with respect to whether it improves survival.*

**What is New:**

*• We have examined predictors of survival in CDH infants including the response to iNO and demonstrated that the highest oxygenation index on day 1 predicted survival (AUCROC =0.908).*

*• Certain infants with CDH responded to iNO and those with a greater response were more likely to survive.*

## Introduction

Infants with congenital diaphragmatic hernia (CDH) can suffer severe morbidity and mortality despite advances in neonatal medicine [1–3]. Pulmonary hypoplasia and pulmonary hypertension are major determinants of mortality in infants with CDH [4]. Pulmonary hypoplasia severity can result in alterations in arterial blood gases [5–7] and this is reflected in a high oxygenation index (OI). The best oxygenation index on day 1 (BOId1) after birth was associated with better survival in CDH infants [8]. We hypothesised that the highest oxygenation index on day 1 (HOId1) would predict survival and an aim of this study was to test that hypothesis.

Inhaled nitric oxide (iNO) is a potent pulmonary vasodilator, however, the efficacy of iNO in treating pulmonary hypertension in infants with CDH has been variable. One study reported that administration of pulmonary vasodilators was associated with better oxygenation and improved survival after ECMO [9]. Another study showed that use of iNO with early surgery was associated with improved outcomes in prenatally diagnosed infants [10]. A further study showed that iNO was associated with improved oxygenation and reduced need for ECMO in CDH infants with pulmonary hypertension and normal left ventricular systolic function [11]. In contrast, a population based study involving 70 centres from 13 countries showed that iNO use was associated with an increased risk of mortality [12]. In a recent Cochrane review, it was recommended not to use iNO in CDH infants with hypoxic respiratory failure [13]. Despite this, there is wide variability in the use of iNO in CDH infants [12]. Indeed, one study demonstrated that 36% of CDH infants without evidence of pulmonary hypertension received iNO therapy [12]. We, therefore, aimed to identify factors associated with survival in CDH infants and whether this was influenced by the response to iNO.

## Methods

### Study subjects

An observational study was conducted in the tertiary medical and surgical perinatal centre at King’s College Hospital (KCH) NHS Foundation Trust, London. Infants with CDH during a 10-year period (01/01/2011 to 01/01/2021) were included in the study. The study was registered with the Clinical Governance and Audit Department of KCH (Registration No:CH096) and did not require informed parental consent.

KCH is a tertiary referral centre for CDH infants who might benefit from the antenatal intervention of fetoscopic endoluminal tracheal occlusion (FETO). Fetuses with left sided CDH received FETO during the TOTAL trial (NCT0240057) and right sided CDH fetuses were offered FETO on compassionate grounds [14]. FETO was performed between 27 and 32 weeks of gestation depending on the severity of the CDH. After birth, infants were intubated and started on “gentle” ventilation as per the CDH EURO Consortium guidelines [15]. Infants that required a peak inflation pressure of more than 28 to 30 cm H_2_O to maintain adequate blood gases were transferred to high frequency oscillation (HFO). Inhaled nitric oxide (iNO) was started if there was pulmonary hypertension as evidenced by either echocardiography or a difference in the pre- and post-ductal saturations of more than 10%. The echocardiographic identification of pulmonary hypertension was done using quantitative and qualitative assessments. Quantitative assessment included the estimation of the ratio of tricuspid regurgitant jet {estimation of right ventricular systolic pressure (RVSP)} to the systolic blood pressure (SBP). A ratio greater than 0.5 was considered as CDH associated pulmonary hypertension. In the absence of tricuspid regurgitation, qualitative assessment was done (i) flattened or left deviated interventricular septum in ventricular systole (ii) right to left shunting across the patent foramen ovale, atrial septal defect or patent ductus arteriosus (iii) right ventricular hypertrophy, dilatation or dysfunction [16]. Inotropic support was considered in infants whose mean arterial blood pressure was lower than the gestational age of the infant. The infants were deemed stable for surgery once the iNO was weaned off and they were on a minimal dose of one inotrope (5 µg/kg/min of dopamine) and their FiO_2_ requirement was less than 0.5. CDH infants with an oxygenation index of 25 despite maximal conventional therapy were discussed with an ECMO centre.

### Data collection

Demographic data were collected from the medical and surgical notes and included – prenatal diagnosis, observed to expected lung area to head circumference ratio at diagnosis, underwent a FETO procedure, mode of delivery, administration of antenatal corticosteroids, side of the CDH, gestational age, birthweight, male sex,, preoperative need for HFO, need for iNO, highest oxygenation index in the first 24 h (HOId1), preoperative need for inotropic support, need for ECMO, surgical repair, thoracoscopic repair, evidence of liver in thoracic cavity, patch repair, mortality before discharge from the neonatal care, ventilation free days (VFD) by day 28 after birth, intensive care free days (ICUFD) by day 28 after birth, need for home oxygen on discharge from neonatal care.

We calculated VFD and ICUFD by day 28 after birth. Ventilation free days were calculated to take into account the impact of morbidities in conditions associated with increased mortality as follows:

- VFD = 0, if the subject died within 28 days of mechanical ventilation.
- VFD = 28 – x, if successfully extubated from mechanical ventilation (x days) from initiation.
- VFD = 0, if the subject is mechanically ventilated for more than 28 days.
- Intensive care free days (ICUFD) was calculated in similar manner [17].

The effect of iNO was determined by calculating the PaO_2_/ FiO_2_ ratio and oxygenation index (OI). The arterial oxygen level (PaO_2_), the fraction of inspired oxygen (FiO_2_) and the mean airway pressure (MAP) prior to and 30–60 min after initiation of iNO were collected to calculate the PaO_2_/FiO_2_ ratio and OI. The OI was calculated using the formula OI = MAP × 100 x FiO_2_/PaO_2_ [18]. The percentage change in PaO_2_/FiO_2_ ratio at the two defined points (prior to and 30–60 min of initiation of iNO) was used to assess the response to iNO.

### Analysis

The data were assessed for normality using the Kolmogorov–Smirnov test which showed the data were non-normally distributed, hence, the data were assessed for statistical significance using the Mann–Whitney U test or Chi-square test. The strengths of the correlations with ventilator free days and intensive care-free days with gestational age, Apgar score at 5 min, O/E LHR and HOId1 were determined by calculating Spearman’s rank correlation coefficients. Multivariable logistic regression analysis was undertaken to identify the effect of HOId1 on survival after adjusting for other variables shown to be significant on univariate analysis (gestational age, Apgar score at 5 min, and the O/E LHR). Multivariable linear regression analysis was undertaken to identify the effect of HOId1 on ventilator free days and intensive care free days after adjusting for gestational age, Apgar score at 5 min, O/E LHR and preoperative use of HFO. Multi-collinearity among the predictor variables in regression analysis was assessed by tolerance in the predictor variable in multi-collinearity statistics. Additionally, the data was checked for multicollinearity and if the predictors had a correlation coefficient of more than 0.7, one of the variables was removed to form a composite variable. The Analysis of Variance (ANOVA) in the model summary (of regression analysis) was used to identify the goodness of fit. All the regression analyses had ANOVA *p* values of < 0.05 and so considered to be a good fit for predicting the dependent outcome. Cases of missing data for any of the variables in the multivariable linear regression analysis were removed list-wise from the model. The strength of correlations of iNO response (percentage change in PaO_2_/FiO_2_ ratio) with gestational age, birthweight, Apgar score at 5 min and O/E LHR were also determined using Spearman’s rank correlation coefficients. To predict the effect of highest oxygenation index and the response to nitric oxide on survival, receiver operating curves (ROC) were constructed and the areas under the curve calculated. An optimal cut-point from the ROC curve was selected which corresponded to the combination of the highest sensitivity and specificity. IBM SPSS Statistics for Windows, Version 27.0 (SPSS Inc. Chicago, IL) was used to analyse the data.

## Results

During the 10-year study period, 105 infants were admitted with CDH. The infants had a median (interquartile range) gestational age of 38^+0^ (34^+4^ – 39^+0^) weeks and birthweight of 2.17 (2.22–3.13) kilograms (Table 1). Twelve (11.4%) infants were out born. Ninety-five infants (90.5%) were diagnosed antenatally; they had a median (IQR) O/E LHR of 30.7 (22–40) and 37 infants underwent a FETO procedure. Forty-six infants (43.8%) infants died; seven died post-operatively, whilst 39 infants died prior to surgery.

**Table 1:** Demographics of the CDH infants. Data expressed in median [interquartile range] or *N* [%]

**Characteristics**	**Median [IQR]**	***N* (%)**
Gestational age (weeks)	38^+0^ (34^+4^–39^+0^)	
Birthweight (kilograms)	2.17 (2.22–3.13)	
Male gender		59 (55.1)
Inborn		93 (88.6)
Prenatal diagnosis		95 (90.5)
O/E LHR	30.7 (22–40)	
Right sided		16 (15.2)
Liver up in thorax		21 (20.6)
FETO done		37 (35.2)
Antenatal steroids (at least 1 dose)		51 (48.6)
Caesarean section		38 (36.2)
High frequency oscillation		57 (54.3)
Surfactant		25 (23.8)
Inhaled nitric oxide		72 (68.6)
Inotropes		91 (86.7)
ECMO		5 (4.7)
Sepsis		14 (13.3)
Surgical repair		66 (62.9)
Day of surgery	4 (3–6)	
Open repair (vs thoracoscopic) *N* = 66		61 (92.4)
Patch repair		24 (36.3)
Mortality		46 (43.8)
Home oxygen at discharge		8 (13.6)
Ventilator-free days	6 (0–20)	
Intensive-care-free days	0 (0–18)	

Infants who survived were more mature compared to those who did not survive (*p* = 0.007) and the five minute Apgar score was significantly higher in infants who survived (*p* < 0.001) (Table 2). Forty-five of the 46 infants who died were diagnosed antenatally (97.8%) and prenatal diagnosis was associated with increased mortality compared to postnatal diagnosis (*p* = 0.03). The CDH infants who survived had a higher median O/E LHR (*p* < 0.001). Infants who died had a higher median HOId1 (*p* < 0.001). On multivariate regression analysis, HOId1 independently predicted survival [adjusted *p* = 0.004, OR 0.948 (0.913–0.983)]. On ROC analysis, an HOId1 of 31 had 90% sensitivity and 80% specificity in predicting death (*AUC* = 0.908).

**Table 2:** Demographics by survival status. Data expressed as median [IQR] or *N* (%)

**Characteristics**	**Survivors** *N* = 59		**Non survivors** *N* = 46	***p* value**
Gestational age(weeks)	38^+4^ (35^+2^–39^+1^)	35^+6^ (34^+0^–38^+4^)		0.007
Birthweight (kilograms)	2.91 (2.34–3.2)	2.49 (1.82–2.97)		0.004
Male gender	36 (61)	23 (50)		0.26
Apgar at 5 min	9 (7–9)	7 (4–8)		< 0.001
Prenatal diagnosis	50 (84.7)	45 (97.8)		0.023
O/E LHR	35.5 (27.9–43.4)	24 (18.4–35.9)		< 0.001
Right sided	8 (13.6)	8 (17.4)		0.59
Liver up in thorax	12 (21.4)	9 (19.6)		0.82
FETO done	16 (31.4)	21 (46.7)		0.12
Antenatal steroids (≥ 1 dose)	23 (39)	28 (60.9)		0.026
Caesarean section	23 (39)	15 (32.6)		0.50
High frequency oscillation	18 (30.5)	39 (84.8)		< 0.001
Inhaled nitric oxide	28 (47.5)	44 (95.7)		< 0.001
Highest OI in day 1	13.8 (6.1–26.5)	74.5 (48.6–114.7)		< 0.001
Inotropes	50 (84.7)	41 (89.1)		0.51
ECMO	3 (5.1)	2 (4.3)		0.86
Sepsis	9 (15.3)	5 (10.9)		0.51

Infants who had FETO had fewer ventilator free-days (VFD) by day 28 after birth [0 (0–10) days) compared to infants who did not have FETO [13 (0–21) days, *p* = 0.005]. Infants who required HFO preoperatively had fewer VFD by day 28 after birth [0 (0–7) days] compared to those who did not require HFO [20 (11–22) days, *p* < 0.001]. A patch repair was associated with fewer VFD [10 (0–17) days] compared to primary repair [21 (19–23) days, *p* < 0.001]. There were significant correlations of gestational age (*r* = 0.426, *p* < 0.001), 5-min Apgar score (*r* = 0.49, *p* < 0.001), O/E LHR (*r* = 0.371, *p* < 0.001) and HOId1 (*r* = − 0.697, *p* < 0.001) with VFD by day 28 after birth. Infants who had FETO had lower ICUFD by day 28 after birth [0 (0–2) days) compared to infants who did not have FETO [10 (0–19) days, *p* < 0.001]. Infants who required HFO preoperatively had lower ICUFD by day 28 after birth [0 (0–2) days] compared to those who did not require HFO [16 (1–20) days, *p* < 0.001]. A patch repair was associated with lower ICUFD [2 (0–9) days] compared to primary repair [19 (11–22) days, *p* < 0.001]. There were significant correlations of gestational age (*r* = 0.485, *p* < 0.001), 5-min Apgar score (*r* = 0.532, *p* < 0.001), O/E LHR (*r* = 0.357, *p* < 0.001) and HOId1 (*r* = − 0.66, *p* < 0.001) with ICUFD by day 28 after birth. The correlations of ICUFD remained significant for O/E LHR (*p* = 0.02) and HFO (*p* = 0.01) after adjusting for gestational age, 5-min Apgar score and HOId1.

Seventy-two infants (68.6%) received iNO; 28 survived to discharge. The median PaO_2_ improved after initiation of iNO (pre 46.7 versus post 58.8 mmHg, *p* < 0.001) and the median PaO_2_/FiO_2_ ratio improved post iNO (pre 49.4 versus post 58.8, *p* < 0.003), but the median OI remained similar (pre 28.6 versus post 26.9, *p* = 0.28). The iNO response was not significantly affected by gestational age (*r* = − 0.036, *p* = 0.77), birthweight (*r* = 0.013, *p* = 0.92), Apgar score at 5 min (*r* = 0.142, *p* = 0.27), O/E LHR (*r* = 0.136, *p* = 0.304) or FETO (*p* = 0.16). The percentage change in PaO_2_/FiO_2_ ratio post iNO initiation was significantly higher in infants who survived (69.4%) compared to infants who died (10.2%, *p* = 0.018). On ROC analysis, a percentage change in PaO_2_/FiO_2_ ratio of 20% on initiation of iNO was associated with 73% sensitivity and 60% specificity in predicting survival (*AUC* = 0.672).

## Discussion

We have demonstrated that survival in infants with CDH is related to highest OI on day 1 after birth. Furthermore, we highlight that the response to iNO can predict survival in CDH infants.

The infants with CDH who survived were more mature, of higher birthweight had higher O/E LHR and had better Apgar score at 5 min. A previous study showed that higher birthweight and Apgar score at 5 min were related to survival [5, 19]. Other studies have shown that lower gestational age is related to increased mortality [20, 21] and the association of O/E LHR to survival has been reported previously [20, 22]. Our study showed that the HOI on day 1 was associated with survival after adjusting for gestational age, Apgar score at 5 min and the O/E LHR. A previous study showed that highest OI was associated with survival [23]. That study, however, assessed the highest OI in first 48 h rather than in the first 24 h as in our study and was limited to infants with left sided CDH. The current study showed that HOId1 was a good predictor of survival in both left or right sided CDH infants with ROC *AUC* of 0.908.

Various factors have been reported in predicting survival in CDH. The Congenital Diaphragmatic Hernia Study Group highlighted birthweight and five minute Apgar score [24] and the Canadian Neonatal Network (CNN) used the SNAPE-II (Score for Neonatal Acute Physiology, Version II) score and birthweight [25]. We have previously demonstrated that non-invasive assessment of the ventilation perfusion ratio is a good predictor of survival with an *AUC* of 0.905 [26]. Schultz et al. used the Wilford Hall/ Santa Rosa prediction formula (highest PaO_2_ – highest PCO_2_) derived from arterial blood gases obtained within 24 h to predict survival with an *AUC* of 0.87 [27]. Amodeo and colleagues calculated the radiographic pulmonary area from the preoperative chest radiograph performed within 24 h of birth in infants with CDH and found that total pulmonary area predicted survival with an *AUC* of 0.801. They also reported that the ipsilateral pulmonary area predicted survival with an *AUC* of 0.772 and the contralateral pulmonary area predicted survival with an *AUC* of 0.775 [28].

Our study showed that CDH infants who required HFO preoperatively had almost no ventilator free days within the first 28 days after birth. A previous study found that ventilator free days by day 60 were significantly lower in infants with severe CDH with pulmonary hypertension compared to those with severe CDH and no pulmonary hypertension [29]. In our institution, HFO is considered in cases where oxygenation and ventilation remain poor despite high airway pressures during conventional ventilation and administration of iNO. This likely explains the association of a paucity of VFD in infants treated with HFO. Intensive care-free days were significantly higher in infants with better O/E LHR at diagnosis. O/E LHR has been reported as a marker of severity of CDH and predictor of outcome in CDH [22]. Our study showed that FETO was not independently associated with fewer ventilator-free days or intensive care free days. This is similar to findings of the TOTAL trial which categorised infants to moderate and severe left sided CDH [30, 31].

The response to inhaled nitric oxide was variable in infants with CDH in our study, but infants who responded to iNO were more likely to survive. The PaO_2_ and PaO_2_/FiO_2_ ratio were significantly improved post iNO initiation in the survivors compared to non survivors. Infants who did not survive had a higher MAP which may have resulted in the lack of a significant difference in the OI results between the survivors and non survivors, despite improvements in oxygenation. Kumar et al. considered an increase in 20 mm Hg of PaO_2_/FiO_2_ post initiation of iNO as a positive response to iNO [9]. Infants who had an increase of 20 mmHg in oxygenation were less likely to require ECMO (24% versus 50%) in one series [11]. Our study showed that an increase in 20% post iNO initiation predicted survival.

Our study has strengths and some limitations. This is one of the few studies which looked into factors affecting ventilator free days and intensive care-free days in infants with CDH We believe it is the first study to report the effect of the response to iNO in predicting survival in infants with CDH. Limitations were the retrospective nature of the study, but we made a comprehensive data collection. Very few infants were transferred for ECMO, so we cannot appropriately comment on whether the response to INO influenced the need for ECMO.

In conclusion, the highest oxygenation index on day 1 of life was a better predictor of survival than gestational age and the Apgar score at 5 min. Inhaled nitric oxide was associated with improved oxygenation in certain infants with CDH and the response to nitric oxide was better in those infants who survived.

## Abbreviations

BOId1: Best oxygenation index on day 1
CDH: Congenital diaphragmatic hernia
ECMO: Extracorporeal membrane oxygenation
FETO: Fetoscopic endoluminal tracheal occlusion
FiO_2_: Fraction of inspired oxygen
HFO: High frequency oscillation
HOId1: Highest oxygenation index on day 1
ICUFD: Intensive care free days
iNO: Inhaled nitric oxide
MAP: Mean airway pressure
OI: Oxygen indices
PaO_2_: Arterial oxygen level
VFD: Ventilation free days
